# “Face of a Giant Panda” and “Beating Wings” in a Young Male

**DOI:** 10.7759/cureus.22429

**Published:** 2022-02-21

**Authors:** Navneet Arora, Kushal Wasti, Vikas Suri, Pankaj Malhotra

**Affiliations:** 1 Internal Medicine, Postgraduate Institute of Medical Education and Research, Chandigarh, IND

**Keywords:** batwing tremors, wilson's disease with primarily neurological presentation, wing beating tremors, face of a giant panda, wilson's disease

## Abstract

Mutations in the gene coding for ATPase copper transporting beta polypeptide (ATP7B) cause Wilson's disease, located on chromosome 13. It has mainly hepatic and neurological presentations. Movement disorders are a characteristic finding in Wilson's disease, and “wing-beating tremors” are classical characteristics found on physical examination. We came across and managed a case of Wilson's disease with primarily neurological presentation with classical wing-beating tremors and “face of a giant panda” on radiology. As the patient had very typical findings and he also improved with the treatment, it will be beneficial to the clinicians in their daily practice to identify the disease seeing these clinical findings.

## Introduction

Wilson's disease presents with hepatic, psychiatric, and neurological manifestations due to impaired copper metabolism and deposition. It has a peak incidence around 17 years of age. However, the onset of presentations in infancy and above 70 years is also well documented [1]. Genetic testing for ATPase copper transporting beta polypeptide (ATP7B) confirms the diagnosis and is increasingly available nowadays. Various studies suggest that the incidence of Wilson's disease is much more common than previously estimated as a result of improved testing facilities [2,3]. Early diagnosis and initiation of therapy improve the quality of life to an extent of eventually reversing the established injury.

## Case presentation

**Figure 1:**
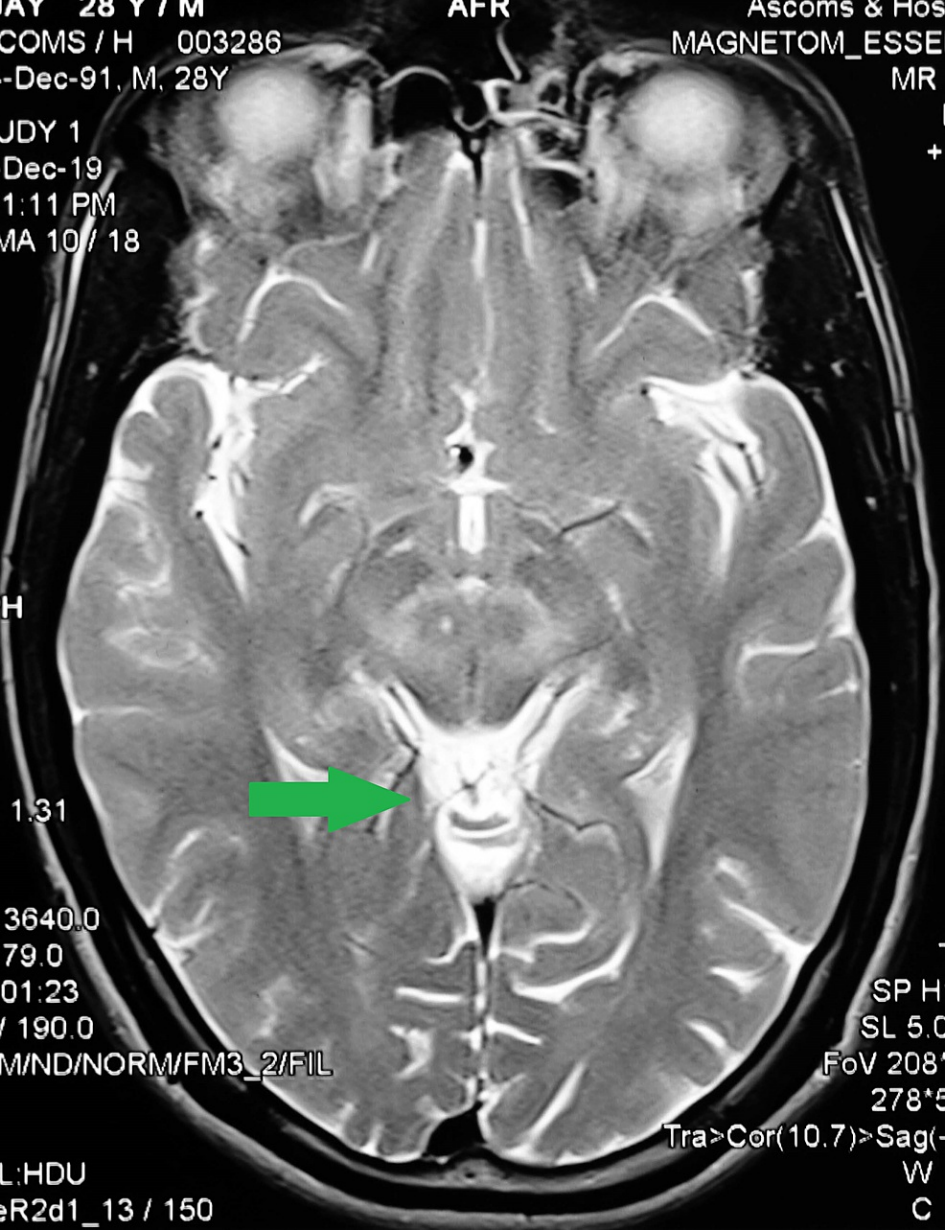
Magnetic resonance imaging (MRI) of the brain revealing T2-weighted high-signal intensities in the caudate nucleus and putamen of bilateral basal ganglia, with sparing of the globus pallidus, ventrolateral thalami, the brainstem, and pontine region, giving an appearance of “face of a giant panda” (green arrow).

A 28-year-old male patient presented to Postgraduate Institute of Medical Education and Research, Chandigarh, India, with involuntary upper and lower limb movements for 18 months. It started from the right arm and gradually progressed to both the upper limbs and lower limbs. He denied alcohol use and reported a 2.5 pack-year smoking history. There was no history of any neurological, psychiatric, or liver disease in the family. There was no history of a decrease in educational performance or any hyperactivity. He denied the use of copper utensils for cooking or eating. On examination, the patient was afebrile and had a pulse rate of 78 beats per minute and a blood pressure of 110/70 mm of Hg. Eye examination revealed a golden brown ring at the periphery of the cornea, Kayser-Fleischer ring. Neurological examination revealed wing-beating tremors over the upper limbs. Tremors were more prominent on the right upper limbs, present at rest, and aggravated by movement (Video 1). The clinical possibility of Wilson's disease with primarily neurological presentation was considered, and he was future evaluated. Initial blood workup showed hemoglobin of 14.2 mg/dL with thrombocytopenia (platelet count of 80,000/ mm^3^). His liver chemistry panel was within normal limits; however, transient elastography (FibroScan) showed elevated hepatic stiffness (liver stiffness measurement of 14.3 kPa), with a low serum ceruloplasmin level of 2.8 mg/dL (normal: 22-58 mg/dL). Brain magnetic resonance imaging (MRI) revealed T2-weighted high-signal intensities in the caudate nucleus and putamen of the bilateral basal ganglia, with sparing of the globus pallidus, ventrolateral thalami, brainstem, and pontine region, giving an appearance of “face of a giant panda” (Figure 1). The patient was started on oral zinc acetate 50 mg thrice daily and was monitored for improvement in symptoms. The patient responded to the given treatment, which resulted in the cessation of his involuntary movements. He was advised to follow up on an outpatient basis. Direct sequencing of ATP7B for his siblings was also planned, and they were also referred to the outpatient department.

**Video 1:** Wing beating tremors over the upper limbs, more prominent on the right side.

## Discussion

Wilson's disease is a hereditary disorder that leads to co-occurrence of liver cirrhosis with neurological deficits that are predominately extrapyramidal. It is caused by a heterozygous mutation in the ATP7B located on chromosome 13 [4-6]. Increase in intracellular copper causes oxidative stress and free radicle formation, leading to cell death in the hepatic and brain tissues [7]. Neurological manifestations in Wilson's disease are typically found in the second or third decade of life. The presence of classic wing-beating tremors or flapping tremors in the presence of dysarthria should lead to a strong suspicion of this condition, though other causes such as metabolic encephalopathies and idiopathic Parkinson’s disease should be excluded. Irregular and jerky dystonic tremors are the most common form of Wilson's disease with primarily neurological presentation. Kayser-Fleischer rings are present in 25-50% of patients with the pre-symptomatic or hepatic disease and in >99% cases of Wilson's disease with primary neurological involvement [8]. Low serum ceruloplasmin level (<100mg/L) and low serum copper along with clinical manifestations are sufficient to establish the diagnosis. Liver biopsy for hepatic copper measurement is rarely indicated in Wilson's disease with primarily neurological presentation. On MRI of the brain, the typical “face of giant panda” is found in only 14.3% of the patients; however, tectal plate hyperintensity, central pontine myelinolysis-like abnormalities, and concurrent signal changes in the basal ganglia, thalamus, and brainstem are the more common findings [9-11]. These findings generally regress with successful treatment. Direct genetic testing of ATP7B gene mutation plays a vital role in diagnosing Wilson's disease. Wilson's disease requires lifelong medical therapy, which is given in two phases: acute de-coppering treatment and subsequent maintenance therapy. Copper chelators (penicillamine, trientine, and tetrathiomolybdate), zinc, or both are used to manage the disease. Family screening of clinically asymptomatic siblings and their parents is a must to detect pre-symptomatic disease. Measurement of copper and ceruloplasmin levels and examination for Kayser-Fleischer rings are minimal; however, genetic testing for all first-degree relatives should be done ideally [12].

## Conclusions

Wing-beating tremors are classical findings in Wilson's disease. The typical “face of a giant panda” is found only in few patients and can regress entirely after treatment. Kayser-Fleischer rings are present in 25-50% of patients with pre-symptomatic or hepatic disease and in >99% cases of Wilson’s disease with primarily neurological presentation. The diagnosis of Wilson’s disease will often require examination of 24-hour urinary copper excretion, ceruloplasmin, liver biopsy, and genetic testing. Copper chelators (penicillamine, trientine, and tetrathiomolybdate), zinc, or both are used to manage the disease, and the patient requires lifelong medical therapy.
